# Risk Factor for Incident Functional Disability and the Effect of a Preventive Exercise Program: A 4-Year Prospective Cohort Study of Older Survivors from the Great East Japan Earthquake and Nuclear Disaster

**DOI:** 10.3390/ijerph15071430

**Published:** 2018-07-06

**Authors:** Yujiro Kuroda, Hajime Iwasa, Masatsugu Orui, Nobuaki Moriyama, Claudia Kimie Suemoto, Chikako Yashiro, Kumiko Matsuda, Seiji Yasumura

**Affiliations:** 1 Department of Public Health, Fukushima Medical University, Fukushima, Fukushima Prefecture 960-1295, Japan; hajimei@fmu.ac.jp (H.I.); oruima@fmu.ac.jp (M.O.); moriyama@fmu.ac.jp (N.M.); yasumura@fmu.ac.jp (S.Y.); 2 Department of Public Health, Iitate Village, Fukushima Prefecture 960-1803, Japan; c-yashiro@vill.iitate.fukushima.jp (C.Y.); k-matsuda@vill.iitate.fukushima.jp (K.M.); 3 Tokyo Metropolitan Institute of Gerontology, Tokyo 173-0015, Japan; 4 Discipline of Geriatrics, University of São Paulo Medical School, São Paulo SP 01246-903, Brazil; cksuemoto@usp.br

**Keywords:** functional disability, Great East Japan earthquake, cohort study, basic checklist, exercise intervention

## Abstract

Objective: The aim of this study is to assess the risk factors for incident functional disability among long-term evacuees of Iitate village after Great East Japan Earthquake and nuclear disaster (GEJE). We also investigated the effectiveness of exercise classes as an intervention measure in this situation. Methods: 1159 subjects (75.2 ± 5.8 years, 57.5% female) were included at baseline, and followed-up for four years. Cox proportional hazard regression analysis was used to estimate the hazard ratio (HR) of incident functional disability according to the presence of risk factors evaluated by the municipality’s self-assessment Basic Checklist (BCL). Evacuees from Iitate who participated in the exercise classes and those who did not were matched using the propensity scores, which were then used to obtain the HR of incident functional disability. Results: New functional disability occurred in 280 (24.2%) participants during the follow-up. Participants who scored negative for the “Physical function” domain in the BCL had a HR of 2.04 (95% CI: 1.54–2.69) for incident functional disability when compared to those who scored positive for this domain. Similarly, the HR for “Cognitive function” was 1.37 (CI: 1.06–1.77), and 1.60 (CI: 1.24–2.08) for “Depression”. Using a Cox proportional hazard regression model, both the group with low-participation in the exercise program and the group with high-participation in the exercise program had a significantly lower rate of incident functional disability compared to those who did not participate at all (HR = 0.27, CI: 0.16–0.46; HR = 0.30, CI: 0.12–0.74, respectively). Conclusions: Pre-disaster BCL domains were useful to identify individuals at risk of functional disability after a major socio-technical disaster. Therefore, this instrument can be used to identify at-risk older adults who would benefit from early exercise programs to prevent incident functional disability.

## 1. Background

The Great East Japan Earthquake (GEJE) and the tsunami on 11 March 2011 and the Tokyo Electric Power Company Holdings (TEPCO) Fukushima Daiichi Nuclear Power Plant disaster caused major population movement in the affected areas by people such as those losing homes to tsunami/earthquake, forced evacuation by government orders, and voluntary evacuation. Studies have examined the various mental and physical issues among the victims and evacuees. Physical symptoms include sleep deprivation, elevated blood pressure, and incidence and worsening of chronic diseases [1,2,3]. Deterioration of the whole-body function due to lack of mobility has been observed mainly among older adults and disabled subjects who experienced evacuation. The pros and cons of evacuation have been discussed, including the possibility that it caused a negative spiral in physical and mental health functions [4,5]. Alternatively, post-traumatic stress disorder (PTSD) symptoms from experiencing the disaster or losing families/friends, depressive moods, anxiety, frustration, increase in anger were the observed psychological effects [6,7].

As Japan has the most aged society in the world, and the most affected areas had a high rate of aging population before the disaster, the elderly population bore the brunt of this disaster. In Japan, the frail elderly may receive care and support once they are certified under Japan’s Long-term Care Insurance System (LTCI). The number of incident functional disabilities registered by the LTCI has been constantly rising, and it reached 4.87 million in April 2010, an increase of approximately 2.69 million in 10 years (123%) [8]. Older adults who are assessed as functionally disabled under LTIC tend to suffer from physical and psychological problems as well as alienation from social activities and relationships, leading to a loss of the will to live, which leads to becoming homebound or bedridden with diminished quality of life. Also, incidence of functional disabilities of elderly people who lived in the community until that point are a frequent cause of admission to a hospital or a nursing home, and the use of long-term care services [9,10]. Thus, preventing elderly people from incident functional disability has been a top policy priority for the Ministry of Health, Labor and Welfare in Japan.

Studies have shown that incident functional disability surged in the most heavily affected regions after the GEJE and the nuclear disaster. A survey conducted in the prefectures of Miyagi, Iwate, and Fukushima one year after the GEJE showed that the coastal areas had higher rate of new disability certification (7.1%) compared to the inland areas (3.7%) or other prefectures (2.8%) [11]. Sosō region of Fukushima prefecture, which includes Iitate, had high number of evacuees due to both the tsunami and the nuclear disaster. The rate of new certification for LTCI in Sosō region jumped from 16.2% to 20.3% from 2010 to 2012. In 2014, the rate of functional disability was 25.7% in Iitate, which was even higher than in other municipalities in the Sosō region [12]. The above suggests that displacement due to disaster could be a cause of higher incident functional disability among the elderly. Thus, it is important to further examine the impact of evacuation on the elderly residents, especially identifying the risk factors leading to incident functional disability, as well as finding effective intervention measures for functional disability.

As there is limited research on the impact of prolonged evacuation on the elderly population, this study focuses on evacuees from the village of Iitate in Fukushima prefecture. Iitate is a village in Fukushima prefecture with a pre-GEJE population of about 6000. Being located outside of the 20 km radius, it was not subject to the immediate evacuation order issued after the nuclear disaster. The government issued the order in April 2011, and the evacuation of the entire village was not completed until July 2011. Before the GEJE, Iitate had a high ratio of elderly residents who lived in multigenerational households. According to a survey conducted by the village office, 65.1% of the households split into more than two residences during evacuation, and the number of elderly persons living alone increased [13]. The elderly residents not only had to experience stress over the disaster and the evacuation, but they also lost the support mechanism they had in the family structure or the close-knit community. In addition, the villagers reported a decrease in physical activities, and this trend was more significant among the elderly than in any other group [13]. Iitate office held exercise class programs to improve physical functions and prevent deterioration of physical health in places such as meeting rooms of the temporary housing where many villagers lived.

This study aims to identify the risk factors that caused incident functional disability among long-term evacuees of Iitate village after the GEJE and nuclear disaster. In addition, the study assessed whether the exercise classes held after the GEJE were effective in reducing the risk of functional disability among the elderly persons who had not been assessed with functional disability.

## 2. Methods

### 2.1. Study Cohort

**Figure 1:**
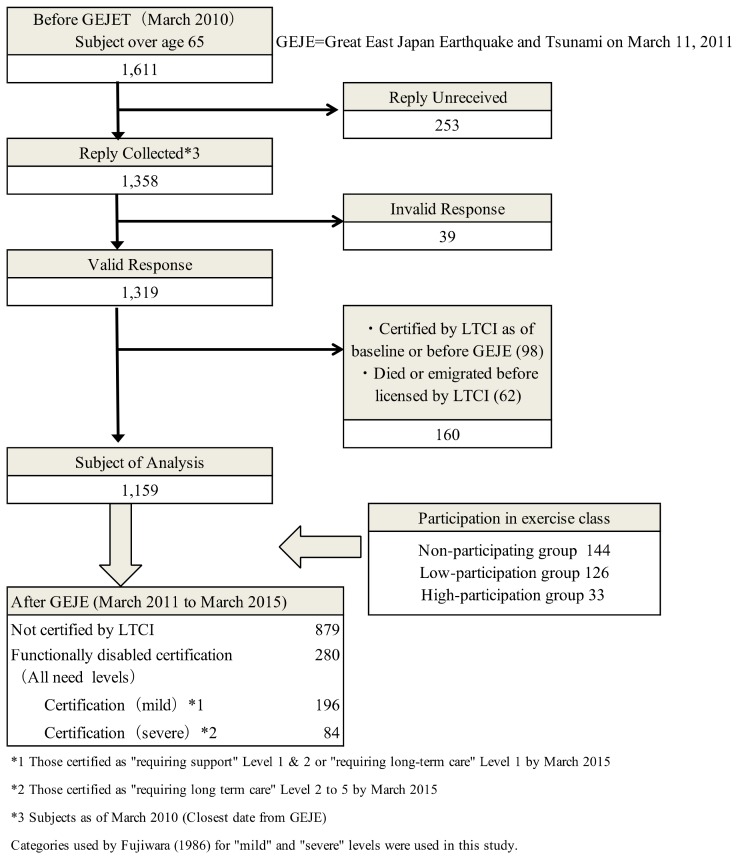
Flow-chart of selection of subject to be analyzed.

This was a prospective cohort study, and in March 2010, a self-assessment ‘Basic Checklist’ (BCL) was distributed to 1611 elderly persons living in Iitate, who were 65 years or older. This baseline survey was conducted as a municipal project to obtain information on residents who were considered to possess high risk of becoming functionally disabled, and Iitate office was responsible for distributing, collecting and analyzing the survey results; 1358 residents responded (response rate of 84.3%). We followed up these residents’ LTCI status until March 2015. We excluded invalid responses (*n* = 39), respondents who had already been certified as disabled by LTCI at the time of baseline survey or prior to the GEJE (*n* = 98), those who died after the disaster without being certified by LTCI, and those who emigrated out of Iitate (*n* = 62). Thus, 1159 subjects were eligible for this study (Figure 1).

### 2.2. Variables

The dependent variable was what we call “incident functional disability”, defined as someone being newly certified by LTCI, regardless of certification level. Under the LTCI, a certification committee in each municipality dispatches a trained investigator to an applicant’s home to evaluate whether they are eligible, then a board consisting of physicians, nurses, and other experts in healthcare and social welfare services determines the levels of support/care by a certification process. There are seven levels of LTCI certification, “requiring support” level 1 and 2, and, “requiring long-term care” level 1 to 5. Based on previous studies by Saito and Fujiwara et al., these were classified into two groups, the “mild” are the “severe”, the former being the “requiring support” level 1 and 2 and “requiring long-term care” level 1, and the latter being “requiring long-term care” level 2 to 5 [14,15]. We removed any person who died or emigrated using information obtained from Iitate office’s database.

As the independent variables, we used the six domains of the BCL developed by the Japanese Ministry of Health, Labor and Welfare (MHLW). BCL is a simple self-reporting yes/no questionnaire consisting of 25 questions regarding instrumental activities of daily living (IADL, three questions), social activities (two questions), physical functions (five questions), nutritional status (two questions), oral function (three questions), homebound (two questions), cognitive function (three questions) and depressive mood (five questions). If a respondent reported having trouble in any of these areas, it is counted as a score in the BCL. It is considered that the higher the score is in a certain domain, the higher the possibility that he/she needs support or care in the said domain. BCL has been used widely among municipalities across Japan, and BCL questions have demonstrated effectiveness in predicting new incident functional disability as well as assessment of frailty [16,17,18]. According to Satake et al., Receiver Operator Characteristic (ROC) curves for the evaluation of frailty status by using BCL was 0.81 for prefrailty and 0.92 for frailty. The sensitivity and the specicity were 70.3% and 78.3 for prefrailty, and 89.5% and 80.7% for frailty at total BCL scores of 3/4 and 7/8 cut-off, respectively [16]. Following a scoring manual published by the MHLW, we considered that a respondent suffered from “dysfunction” in a certain domain from the BCL score for each domain as follows: ‘Physical function’, ≥3 of 5 items; ‘Nutrition’, 2 of 2 items; ‘Oral function’, ≥2 of 3 items; ‘Homebound’, 1 of 1 item; ‘cognitive function’, ≥1 of 3 items; and ‘Depressive moods’, ≥2 of 5 items.

We also obtained additional information from Iitate’s database on residents’ demographics (age, and gender), and baseline medical history (hypertension, heart disease, diabetes, osteoporosis, stroke, hyperlipidemia). Three questions from BCL were used to assess instrumental activities of daily living (IADL). Participants were asked to answer “yes” or “no” to the following questions: “Do you go out by bus or train by yourself?”, “Do you go shopping to buy daily necessities by yourself?”, and “Do you manage your own deposits and savings at the bank?”. For the social domain evaluation, participants were asked to reply in the same manner to two questions from BCL: “Do you sometimes visit your friends?” and “Do you turn to your friends or family for advice?”.

As for evaluating the effect of participation in exercise classes, we calculated the attendance rate for each participant, as well as the average attendance rate of all participants in all locations, then used this average, 45%, as the cut-off rate to obtain the high-participation group, low-participation group and the non-participating group. In other words, any person whose participation rate was no less than 45% was classified in the high-participation group, someone whose rate was less than 45% in the low-participation group, and those who did not participate in the non-participating group. This method was adopted because the intervention effect of participating in the exercise class was considered to be different for frequent participants, less frequent participants and those that did not participate at all.

### 2.3. Exercise Class Program

From April 2012 to March 2013, one-hour long exercise classes were held twice a month in the meeting rooms of the temporary and public housing where evacuees from Iitate lived. Classes continued throughout the year, and participants’ physical fitness was measured at the beginning and end of the year. Sports instructors developed manuals for exercise programs, and carried out various exercises including muscle strengthening, balance improvement, and walking exercise appropriate for the participants’ fitness level [19]. As many elderly residents had to spend long hours in the confined space of their units in the temporary housing, the classes taught exercises that the participants could try at home. As these residents suffered from fewer opportunities to get together in evacuation, recreation activities were also taught so participants could enjoy group interaction. The number of participants varied from 5 to 15, according to the size of the venue. Any evacuee from Iitate could participate in these classes; however, only the data of those above age 65 were used for this study. The village public health nurse and the caretaker of the temporary housing created flyers about the classes to invite the residents.

### 2.4. Statistical Analysis

Baseline characteristic was compared between those certified as eligible for LTCI and those ineligibles for LTCI. We used age, IADL and social domain as continuous variables and conducted unpaired *t*-tests; gender and chronic condition were used as categorical variables for chi-squared test. The frequency of participants’ scores in each of the six BCL domains were converted to “dysfunction at baseline” or “no-dysfunction at baseline” based on the scoring manual mentioned above, which was then compared with their incident functional disability.

Next, a univariate Cox proportional hazard model was conducted with the time (days) until functional disability incidence (any certification) as the dependent variable, and the participant status in each BCL domain as dependent variable. In addition, multivariable Cox proportional hazard regression analysis was conducted, with adjustment for age, gender, chronic condition (a participant falls into this category if he/she has chronic condition for either heart disease, diabetes, osteoporosis, stroke, hyperlipidemia), IADL, and social domain. In addition, the same was conducted for the participants who were evaluated and received “severe” certification as the dependent variable. As LTCI evaluation is conducted by the municipalities, and because the data used in this study was obtained from Iitate village, there was no case where we lost track of the participants.

Furthermore, we investigated whether participation in the exercise classes had any association with incident functional disability. It should be noted that participants of exercise classes were more likely to be more robust than the non-participating group. In other words, participation in the exercise classes designed as an intervention program itself is related to selection bias. To address this issue, we matched both groups using propensity score. To calculate the propensity score, age, gender, chronic condition (the five aforementioned diseases), physical function and depressive mood from BCL domain were used as the predictor variables to estimate the probability of participation in the exercise classes by using logistic regression. Next, samples that had similar propensity score from participating and non-participating groups were matched at a rate of 1:1 to create a data set of 159 people in those two groups. Furthermore, we subdivided the participating group into low-participation and high-participation groups. Then, we carried out a Cox proportional hazard regression analysis with the incidence of LTCI certification as the dependent variable, and participation to exercise classes as explanatory variables. All statistical analyses were conducted with SPSS Statistics for Macintosh, Ver. 24.0 software.

### 2.5. Ethical Issues

The study was conducted as part of the public health projects of Iitate village. The municipal staff working for the project orally explained the research purpose at the time of the health check-up and obtained consent in writing. This study was also approved by the ethics committee of Fukushima Medical University for both baseline data and follow-up data for analysis (Approval No. 2609).

## 3. Results

### 3.1. Incident Functional Disability and Related Risk Factors

During the 4-year follow-up period after the disaster, 280 cases of incident functional disability were registered (24.2% of the sample). Of these, 196 people (70%) were certified as mild, and 84 people (30%) were severe (Figure 1). In the first year, 110 people (out of 280, 39.3%) were assessed with functional disability, in the second year 64 people (cumulatively 174/280, or 62.1%), in the third year 59 people (cumulatively 233/280, or 83.2%), and in the fourth year 47 people (280/280, 100%).

The average age when the subjects were certified with functional disability by the LTCI criteria was 79.5 ± 5.8 years old, and the majority were women (61.1%). In comparison with the group that were not certified, those in the group with functional disability were older and had a higher ratio of women. The most common chronic condition among those assessed with functional disability was hypertension 128 cases (45.7%), and the next was heart disease, with 39 (13.9%) (Table 1). The scores for IADL and social domain were higher in subjects without functional disability than those with functional disability. Increase in age was related to the incidence of functional disability (χ^2^ = 213.96, *p* < 0.001). Among the chronic condition, only osteoporosis was significantly related (χ^2^ = 5.03, *p* = 0.036). People who participated in the exercise classes numbered 159 (13.7%), compared to 1000 non-participants (86.3%).

### 3.2. Association between Basic Checklist (BCL) Domains and Incidence of Functional Disability

Next, incident functional disability (any certification) was compared to BCL domains (Table 2a). 117 people (45.3%) who scored negative for the physical function domain prior to the disaster were assessed with functional disability, whereas 163 people (18.1%) out of 901 who scored positive in this domain at baseline were assessed with functional disability (Crude HR = 3.09, 95% CI = 2.44–3.93). For “oral function”, the incidence of functional disability among those who scored negative in this domain was 31.3%, against 22.7% of those who did not (Crude HR = 1.45, 95% CI = 1.09–1.92). For homebound, they were 38.1% and 21.2% (Crude HR = 2.04, 95% CI = 1.57–2.65), for cognitive function was 31.5% and 18.9% (Crude HR = 1.86, 95% CI = 1.47–2.35), and for “depressive mood”, 35.9% and 19.6% (Crude HR = 2.07, 95% CI = 1.63–2.62), respectively.

In the cases of subjects who received “severe” certification, 34 people (13.2%) who scored negative for the physical function domain prior to the disaster were later assessed with functional disability compared to 50 people (5.5%) out of 901 in the case of those who scored positive in this domain at baseline (Crude HR = 2.92, 95% CI = 1.89–4.52). Similarly, the incidence of functional disability among those who scored negative in the “oral function” domain was 9.5%, against 6.8% of those who did not (Crude HR = 1.46, 95% CI = 0.87–2.43). They were 12.4% and 6.2% (Crude HR = 2.27, 95% CI = 1.42–3.63) for “homebound” domain, 9.9% and 5.3% (Crude HR = 2.07, 95% CI = 1.35–3.20) for cognitive function, and 11.0% and 5.8% (Crude HR = 2.15, 95% CI = 1.40–3.31) for “depressive mood”, respectively.

**Figure 2:**
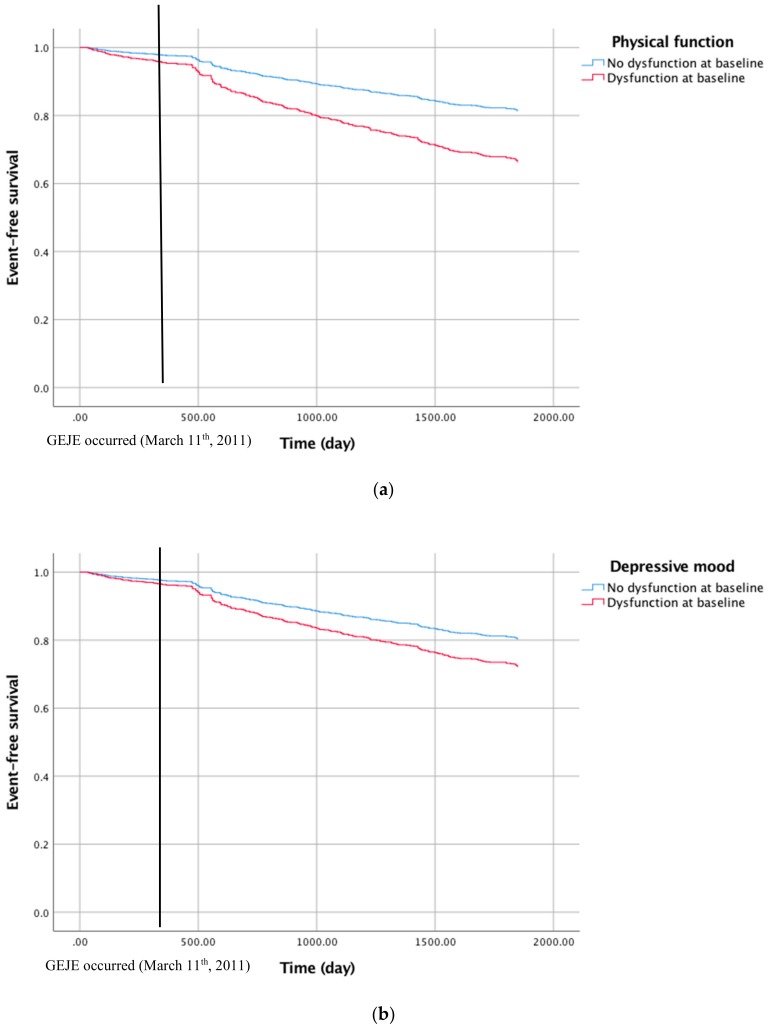
Kaplan–Meier survival curves calculated for all participants including those who were excluded from the analysis (*n* = 98) showing event-free survival to the incident functional disability related to physical function (**a**) and depressive mood (**b**).

Furthermore, in the multivariable Cox proportional hazard model analysis, the HR of incident functional disability among those who scored negative in a certain domain compared to those who scored positive was 2.04 (CI: 1.54–2.69) for “physical function”, 1.37 (CI: 1.06–1.77) for “cognitive function”, and, 1.60 (CI: 1.24–2.08) for “depressive moods”. On the other hand, the result for multivariate analysis, showed that there was no difference regarding oral function or being homebound. We also provided figures of event-free survival of participants (Figure 2).

### 3.3. Intervention Effect of Exercise Classes on Incidence of Functional Disability

**Figure 3:**
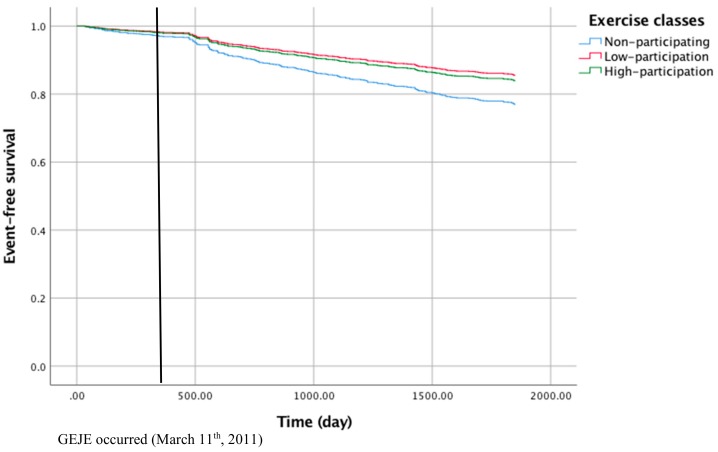
Kaplan–Meier survival curves depicting event-free survival to the incident functional disability related to participation in the exercise classes, including the data of those who were excluded from the analysis (*n* = 98).

Association between exercise classes and incidence of functional disability was analyzed by matching the group that participated in these classes (the group with low participation in the exercise program shall be referred to as the “low-participation” group, those with high participation as the “high participation” group, respectively) with those who did not (the “non-participating” group) using the propensity score method. All differences including age, gender, chronic condition, physical function and depression mood were not significant among three groups after being matched. Using a Cox proportional hazard regression model, both the low-participation group as well as the high-participation group had a significantly lower rate of incident functional disability compared to the non-participating group (HR = 0.27, CI: 0.16–0.46; HR = 0.30, CI: 0.12–0.74, respectively) (Table 3). We also provided figures of event-free survival of participants in exercise classes (Figure 3).

## 4. Discussion

The aim of this study is to assess the risk factors for incident functional disability among long-term evacuees of Iitate village after the GEJE. An additional aim of this study is to analyze the effectiveness of exercise classes conducted after the evacuation in reducing said risk. Using resident data of Iitate village, both before and after the GEJE, we analyzed data of both participants and non-participants of these classes.

This study was not a randomized clinical trial; however, the results suggest a relationship between participation and functional disability, and it may be possible to consider that the exercise classes had some mitigating effect on the incident risk of functional disability. Previous research showed scientific grounds for the health effect of physical activity in alleviating obesity and cardiovascular diseases, preventing the decrease of physical function and falls, and improving depressive symptoms and deterioration of cognitive function [20,21]. There is limited evidence on the group-type intervention through exercise for the people affected by the GEJE. There is a report from Ishinomaki city that described that sense of well-being and the frequency of going out increased significantly after exercising, while mental stress and sleep disorder did not improve. This study concluded that exercise was an effective intervention measure to improve a sense of well-being in disaster-hit areas [22]. However, we must bear in mind that the residents of Ishinomaki city suffered from devastation by a tsunami, so the nature of their experiences may not be directly comparable to those of the evacuees from Iitate. Incident functional disability is an objective index, which is highly relevant for the quality of life of the elderly. The finding of this study suggests that participation in exercise classes could lead to lower incidence of functional disability, although the statistical significance of the mitigation effect and the degree of its effectiveness need to be confirmed by further analyses.

Among the 1159 subjects followed-up for four years after the GEJE, there were 280 (24.2%) cases of incident functional disability. A previous study identified the incident rate of functional disability in those over 65 years after 24 months as 4.5% [23], 8.6% after 36 months [24], and, 8.0% after 40 months [15]. Another study tracking those over 70 years old estimated the rate of 11% with an average follow-up period of 36 months [25]. Compared to these previous studies, the subjects of our study had a higher rate of incident functional disability. It should be noted that the participants of this study underwent confusion and anxiety over the evacuation order, and also experienced long-term evacuation, often accompanied with household breakup and loss of community support. These conditions created by the forced evacuation of the entire village would have contributed to the higher rate. The residents could have had comorbidity with PTSD, and the above conditions could have been symptoms of PTSD and not merely reactions to these conditions; however, we have not evaluated PTSD in our research design so this point cannot be confirmed.

Comparing the degree of physical disability with previous studies, 70% of our subjects were certified as “mild” and 30% as “severe”, while those in Saito et al. were 42.5% and 57.5%, respectively [14]. Therefore, our subjects were more likely to be certified as suffering a “mild” level of disability. Another cohort study analyzed risk factors for mild and severe disability certification [15] and identified that age and the decline of walking ability were related to “mild” disability, whereas age and lack of independence in IADL were related to severe disability [15]. The findings by Fujiwara et al. suggest that people whose walking ability declined due to changes in lifestyle by prolonged evacuation are good candidates for prevention measures.

Residents experienced deterioration of muscle strength, physical strength, and walking ability because they had less opportunity to go out of their houses as they suffered from huge changes in living environment and family composition, as well as being separated from the community they belonged to before the disaster [13]. Another factor could be that before the disaster, people who would have qualified for disability by LTCI were taken care of by families and neighbors. Evacuation increased household separation, so these people now must rely on services provided by the government, which could be a social cause of the increase in incident functional disability.

We also found that post-disaster incident functional disability was higher in the first year (110, or, 39.3%) compared to subsequent years. Evacuation is not just a change of residence. Evacuees must find new ways of transportation, places to shop, clinics to visit, and rebuild human connections, often by themselves. This is highly stressful for elderly people who have lived long in the close-knit community of Iitate, often without any experience of living outside. The disruption in daily lives during the first year would have been the most severe, and this may have led to the decline in functional ability for daily activities among the participants, who were later assessed as having functional disability.

When we compare the baseline risk factors of functional disability of the participants with the pre-disaster data from Miyagi prefecture, the study participants had a lower correspondence rate in all 6 BCL domains before the GEJE. In fact, the difference was larger than 10 points in physical function, cognitive function, and depressive mood. This suggests that the participants were not a high-risk group for functional disability before the GEJE. Another finding is that those who scored positive for physical function, cognitive function, or depressive mood had higher rate of incident function disability after the GEJE compared to those who did not. Stuck et al. have studied predictive factors for functional disability and deterioration of functions, and identified age, medical history, history of falls, BMI, masticatory ability, frequency of going out and depressive mood as high-risk factors for incident functional disability [24,26]. The results of this study support those of previous studies except for “nutritional state”. According to a survey of all Iitate households conducted in 2012, the residents responded with a “decline in physical activities, such as farming (61.9% of total, 71.3% of respondents in 60s) [13]. Before the evacuation, many residents regularly engaged in physical exercise through farming, which was significantly difficult to continue during evacuation. Thus, physical activities decreased significantly especially among the elderly in the evacuation. In another survey conducted by Iitate office, the residents reported “less opportunity to see friends” and a “higher tendency to be irritated”. Life after evacuation is likely to have had negative effect on psycho-social aspects of the residents’ lives, which may have led to a higher rate of incident functional disability.

The above findings suggest that BCL data before the disaster may be useful to predict post-disaster cases of incident functional disability, and participating in exercise classes may be effective in mitigating the incident risk of functional disability. The higher incidence of functional disability in the first year suggests the importance of starting intervention measures early. The takeaway from our study is that, in emergency situations such as post-disaster, early identification of elderly residents at risk, that is, those whose function for daily living has deteriorated, and implementation of intervention such as exercise classes may be effective to reduce the risk of functional disability among the affected population.

### Limitations

As described earlier, this study looked at the cohorts of all the elderly residents of a certain area before and after the GEJE. Our methodology was limited by the following factors. First, as it is not a comparison of randomly chosen samples, the possibility of bias or confounder cannot be denied. Various groups aiming to support the residents have been active in the location of this study, so it cannot be denied or confirmed whether the participants of the exercise class could have been supported by and benefited from said activities. To address this problem, the study aimed to match personal characteristics between the participant group and non-participant group using propensity score matching, although the availability of data limits the statistical adjustment. The second limitation is the lack of control group in considering the risk of requiring care from LTCI, because the entire village had to evacuate due to the government’s order, except for 107 residents of the nursing home for elderly persons, the “Iitate home”, who remained because they were considered highly vulnerable to relocation risk. So, it is not possible to compare groups that evacuated and that did not. The third limitation is that we could not conduct quantified evaluation of how effective the increase in physical exercise from participating in the exercise classes was as an intervention measure. The fourth limitation is that certain important variables related to functional disability, such as PTSD, were not evaluated in our study. Finally, the contents of the exercise programs were not strictly standardized, due to the variation of the participants’ health status and the size of the rooms used for the classes in the temporary housing, among others. Thus, we could not specify which kind of exercise was effective as an intervention.

## 5. Conclusions

The prospective cohort study identified that the BCL domains physical function, cognitive function, and depression before disaster could be risk factors for incident functional disability after a disaster. This study was not a randomized clinical trial; however the results suggest a relationship between participation and functional disability, and it may be possible to consider that the exercise classes had some mitigating effect on the incident risk of functional disability. Another suggestion was that the number of incident functional disabilities was higher in the first year than in subsequent years. Together, the findings suggest that identifying older adults whose functions for daily activities deteriorated as a result of emergencies, such as a natural disaster, at an early stage and implementing intervention measures, such as exercise classes, may be effective in reducing the number of incident functional disabilities, as well as preventing further deterioration of their conditions. For this purpose, pre-disaster BCL may be useful in identifying the targets for intervention.

## Figures and Tables

**Table 1:** Individual characteristics at baseline according to the presence of functional disability (*n* = 1159).

Basic Characteristics	Categories	No Functional Disability *n* = 879	Functional Disability *n* = 280	*p* Value
Age (mean ± standard deviation (SD))		74.0 ± 5.2	79.5 ± 5.8	<0.001
Age group	65–69 (*n* = 231)	215 (24.5)	16 (5.7)	<0.001
	70–74 (*n* = 337)	298 (33.9)	39 (13.9)	
	75–79 (*n* = 317)	237 (27.0)	80 (28.6)	
	80–84 (*n* = 199)	109 (12.4)	90 (32.1)	
	85 and above (*n* = 75)	20 (2.3)	55 (19.6)	
Gender	Male (*n* = 493)	384 (43.7)	109 (38.9)	0.166
	Female (*n* = 666)	495 (56.3)	171 (61.1)	
Chronic condition	Hypertension (*n* = 505)	377 (42.9)	128 (45.7)	0.769
	Heart disease (*n* = 136)	97 (11.0)	39 (13.9)	0.201
	Diabetes (*n* = 91)	70 (7.9)	21 (0.8)	0.095
	Osteoporosis (*n* = 47)	29 (0.3)	18 (0.6)	0.036
	Stroke (*n* = 35)	25 (0.3)	10 (0.4)	0.364
	Hyperlipidemia (*n* = 20)	14 (0.2)	6 (0.2)	0.600
Instrumental activities of daily living (IADL) *^1^		2.5 ± 0.8	2.1 ± 1.1	<0.001
Social domain *^2^		1.8 ± 0.5	1.6 ± 0.7	<0.001

*^1^ IADL (Score range 0–3): Higher scores indicates better the function. *^2^ Social Domain (Score range 0–2): Higher scores indicates better the function.

**Table 2:** (**a**) Incidence of functional disability (Long-term Care Insurance System (LTCI) Certification: any) stratified by Basic Checklist (BCL) domain. (**b**) Incidence of functional disability (LTCI Certification: severe) stratified by Basic Checklist (BCL) domain.

(a)												
Risk Factors	No Dysfunction at Baseline				Dysfunction at Baseline				Univariate Analysis		Multivariate Analysis ^c^	
	No. of Responses		LTCI Certification (Any)		No. of Responses		LTCI Certification (Any)					
	No. of Subjects	% ^a^	No. of Subjects	% ^b^	No. of Subjects	% ^a^	No. of Subjects	% ^b^	Crude Hazard Ratio (HR) (95% CI)	*p* Value	Adjusted HR (95% CI)	*p* Value
Physical function *^1^	901	77.7	163	18.1	258	22.3	117	45.3	3.09 (2.44–3.93)	<0.001	2.04 (1.54–2.69)	<0.001
Nutrition *^2^	1156	99.7	280	24.2	3	0.3	0	0.0	-	-	-	-
Oral function *^3^	958	82.7	217	22.7	201	17.3	63	31.3	1.45 (1.09–1.92)	0.010	1.09 (0.81–1.48)	0.563
Homebound *^4^	957	82.6	203	21.2	202	17.4	77	38.1	2.04 (1.57–2.65)	<0.001	1.18 (0.85–1.65)	0.318
Cognitive functions *^5^	676	58.3	128	18.9	483	41.7	152	31.5	1.86 (1.47–2.35)	<0.001	1.37 (1.06–1.77)	0.015
Depressive moods *^6^	833	71.9	163	19.6	326	28.1	117	35.9	2.07 (1.63–2.62)	<0.001	1.60 (1.24–2.08)	<0.001
**(b)**												
**Risk Factors**	**No Dysfunction at Baseline**				**Dysfunction at Baseline**				**Univariate Analysis**		**Multivariate Analysis ^c^**	
	**No. of Responses**		**LTCI Certification (Severe)**		**No. of Responses**		**LTCI Certification (Severe)**					
	**No. of Subjects**	**% ^a^**	**No. of Subjects**	**% ^b^**	**No. of Subjects**	**% ^a^**	**No. of Subjects**	**% ^b^**	**Crude HR (95% CI)**	***p* Value**	**Adjusted HR (95% CI)**	***p* Value**
Physical function *^1^	901	77.7	50	5.5	258	22.3	34	13.2	2.92 (1.89–4.52)	<0.001	2.11 (1.24–3.58)	0.006
Nutrition *^2^	1156	99.7	84	7.3	3	0.3	0	0.0	-	-	-	-
Oral function *^3^	958	82.7	65	6.8	201	17.3	19	9.5	1.46 (0.87–2.43)	0.149	1.03 (0.58–1.81)	0.925
Homebound *^4^	957	82.6	59	6.2	202	17.4	25	12.4	2.27 (1.42–3.63)	0.001	1.38 (0.75–2.52)	0.299
Cognitive functions *^5^	676	58.3	36	5.3	483	41.7	48	9.9	2.07 (1.35–3.20)	0.001	1.64 (1.01–2.65)	0.046
Depressive moods *^6^	833	71.9	48	5.8	326	28.1	36	11.0	2.15 (1.40–3.31)	0.001	1.68 (1.04–2.72)	0.035

^a^ Ratio against all subjects (*n* = 1159) (%); ^b^ Ratio against the number of respondents (%); ^c^ Cox proportional hazard model adjusted for age, gender, IADL, disease under treatment and Social Domain as adjustable variables for each item above (every item) (Analyzed by Cox proportionate hazard model, “does not fall into category” is the standard); *^1–6^ We considered a domain to indicate “Dysfunction” if the responses were as follows: ‘Physical function’, ≥3 of 5 items; ‘Nutrition’, 2 of 2 items; ‘Oral function’, ≥2 of 3 items; ‘Homebound’, 1 of 1 item; ‘cognitive function’, ≥1 of 3 items; and ‘Depressive moods’, ≥2 of 5 items.

**Table 3:** Relationship between participation in exercise class intervention and incidence of functional disability.

Categories	*n*	Functional Disability		HR	95% CI
		+	−		
Non-participating *^1^	144	60	84	1.00	-
Low-participation	126	18	108	0.27	0.16–0.46
High-participation	33	5	28	0.30	0.12–0.74

*^1^ We excluded 15 participants in Non-participating group that were certified by LTCI before GEJE.
